# Millimeter wave radar data of people walking

**DOI:** 10.1016/j.dib.2020.105996

**Published:** 2020-07-05

**Authors:** Ennio Gambi, Gianluca Ciattaglia, Adelmo De Santis, Linda Senigagliesi

**Affiliations:** Department of Information Engineering, Università Politecnica delle Marche, Ancona, Italy

**Keywords:** mmWave radar, Walking analysis, Automotive radar, Machine learning, Human gait analysis

## Abstract

This dataset contains complex signals coming from a mmWave FMCW radar system. Signals were acquired during a measurement campaign taken indoor and aimed to assess people's different ways of walking. Measurement setup and devices are described.

The dataset consists of the acquisitions of six different types of activities, performed by 29 subjects who repeat each activity several times. Therefore, the dataset contains multiple different experiments for each activity, for a total of 231 acquisitions. The subjects walk without any constraint or do not follow any pattern, thus making this dataset useful not only for human gait recognition but also to evaluate the performance of different radar data processing algorithms.

Specifications Table

**Table:** 

**Subject**	Biomedical, Electrical and Electronic Engineering
**Specific subject area**	Millimeter wave radar acquisitions of different types of walking for their classification
**Type of data**	Matlab data files (.mat)
**How data were acquired**	mmWave RADAR (Texas Instruments AWR 1642)
	Acquisition Board (Texas Instruments DCA 1000 EVM FPGA)
	mmWave Studio (Texas Instruments control software)
	Matlab conversion Script
**Data format**	Raw (from acquisition)
**Parameters for data collection**	Data were acquired on a total of 19 healthy white Caucasian subjects (age: 22.50 ± 1.57 years; height: 173 ± 10 cm; weight: 62.80 ± 9.52 kg) who repeated the activities of Slow walking, Fast walking and Slow walking with hands in pockets for 3 times, for a total of 168 different acquisitions.
**Description of data collection**	During the experiments the subject walks away from the radar along a path orthogonal to the antennas plane. After walking for about half of the acquisition time duration, the subject under test inverts his path re-approaching the radar. The total acquisition duration for each walk is 16 s while the maximum distance is about 10 m from the radar. The subjects were asked to walk normally, without any specific condition to be respected.
**Data source location**	Department of Information Engineering, Università Politecnica delle Marche, Ancona, Italy
**Data accessibility**	Repository name:
	mmWaveRadarWalkingDataset_part1
	https://doi.org/10.5281/zenodo.3824534
	mmWaveRadarWalkingDataset_part2
	https://doi.org/10.5281/zenodo.3897234
**Related research article**	Senigagliesi, L.; Ciattaglia, G.; De Santis, A.; Gambi, E. People Walking Classification Using Automotive Radar. *Electronics* 2020, DOI:10.3390/electronics9040588 (only Repository part 1)

## Value of the data

• These data can be useful to improve algorithms on micro-Doppler analysis for human gait classification.
• The academic community working on radar signal processing and applying artificial intelligence algorithms for human walking classification can significantly benefit from this raw data.
• These data can be used to develop or test algorithms on the beat signal coming from the radar system.
• The dataset is built without issuing any constraint to the subjects. They perform the activities in a natural way so that the dataset is not built ad hoc for the analysis.
• The dataset provides a realistic collection of measurements done on subjects with a large variety of characteristics (age, height, weight) and behaving naturally. It can be used for different analyses on human movements, such as walking speed, hands positions, etc.

## 1 Data description

The dataset provides data from mmWave Frequency Modulated Continuous Wave (FMCW) radar used during a measurement campaign. Tests are performed on walking people who walk in different ways in front of the radar system along a hallway without any furniture or obstacle.

**Fig. 1:**
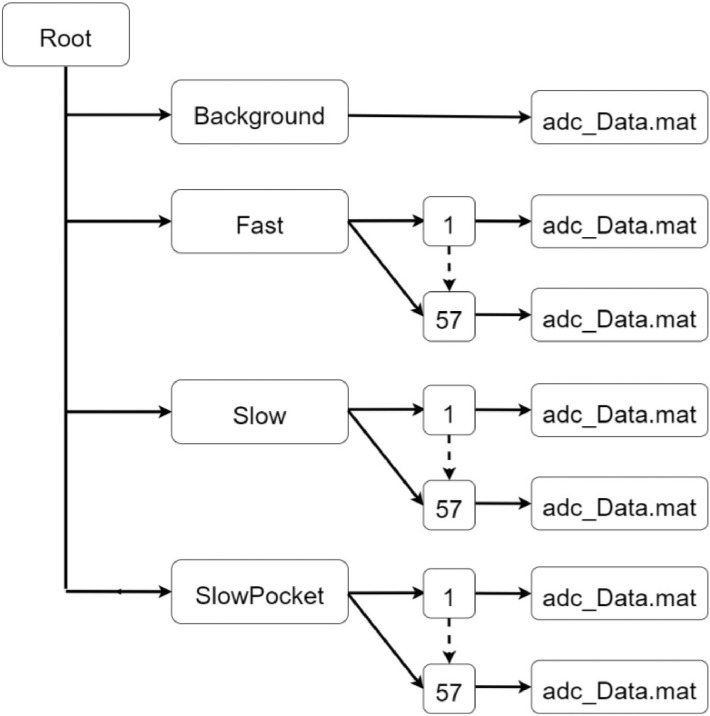
Data structure of the first repository (Part 1).

The repository is divided into two parts. The first one contains all the data collected during the experiments described in [1]. Fig. 1 depicts the Data structure and its content, that will be better described in Section 1.1.

**Fig. 2:**
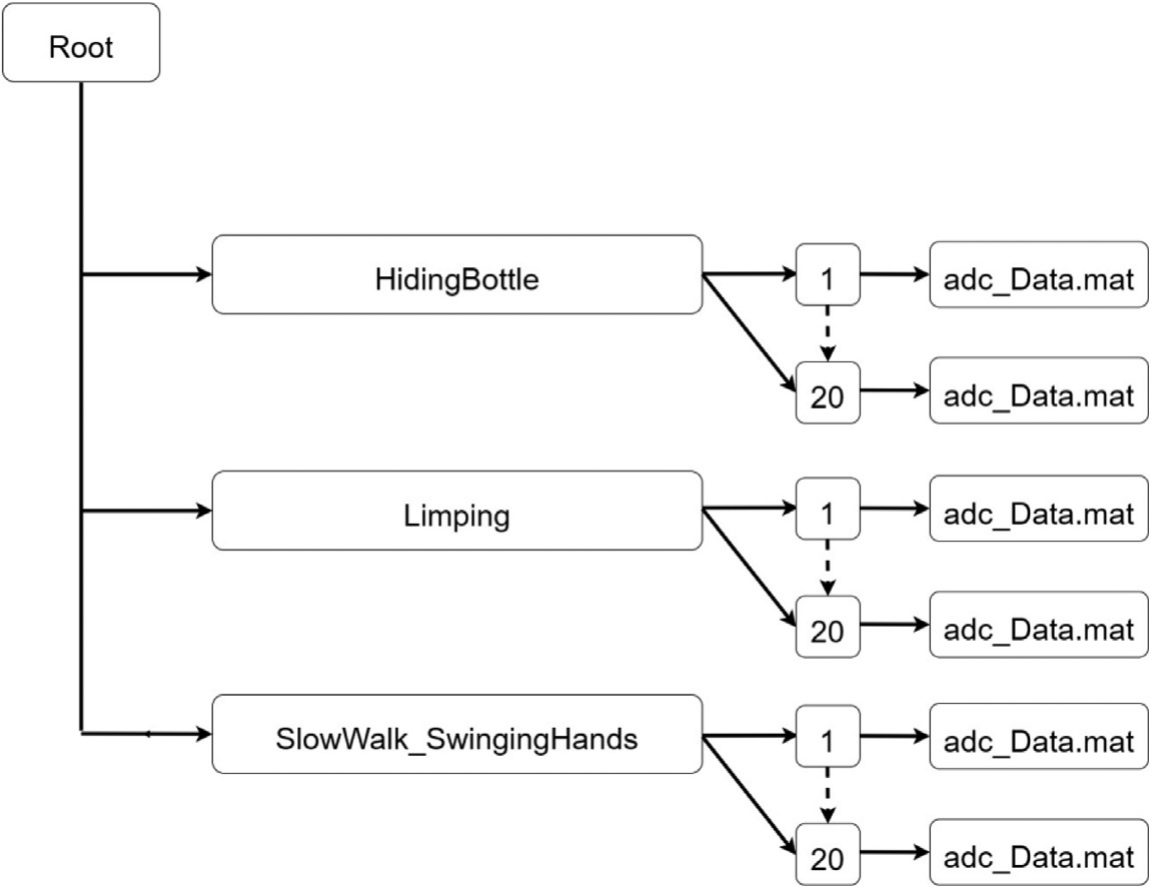
Data structure of the second repository (Part 2).

The second part of the dataset contains data related to different activities, performed in the same test area, which have not been considered in [1]. The data structure is depicted in Fig. 2.

### 1.1 Data repository

The repository is composed of two parts having the same structure. The data structure is composed of a folder for each activity type and background.

In the first repository (Part 1), data from three activities are stored: fast walking is stored in the folder called “Fast”, slow walking in “Slow” and slow walking with hands inside pockets in “SlowPocket”. Inside each activity folder, there are 57 folders which contain data from each test. Each activity was repeated three times by the 19 different subjects, thus leading to 57 different folders.

In Table 1 a summary of all the experiments is reported.

**Table 1:** Details of the experiments contained in dataset_Part1.

Activity	N° of subjects	N° of repetitions	Total N° of tests
Fast	19	3	57
Slow	19	3	57
Slow Pocket	19	3	57

A “.mat” file is contained inside each experiment folder. This file is called “adc_Data.mat” and has the same name for all the tests.

The “background” folder contains data collected from the background, i.e. the radar trace of the environment in which tests were performed. Being the test location always the same, we have only one file.

**Fig. 3:**
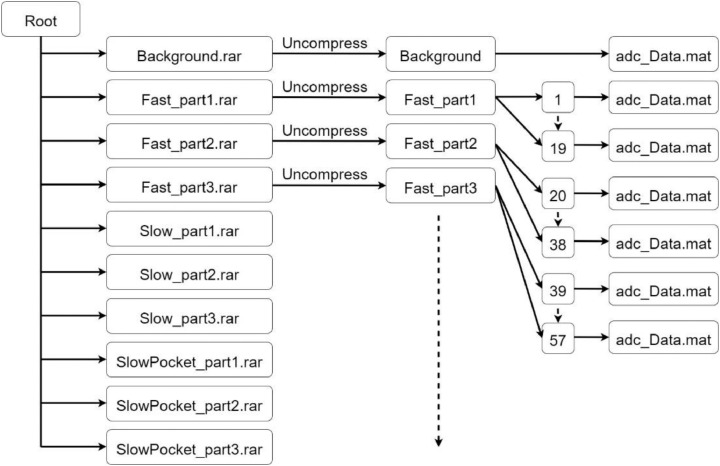
Repository Part 1 structure.

The dataset_Part1 occupies about 73 GB. For reasons of storage, the folders related to each activity have been divided into 3 compressed files. In the repository, the structure of the dataset is then made up of 10 .zip files, as shown in Fig. 3.

As Part 1, also the repository Part 2 contains data from three different activities. These are stored in a folder with the name “HidingBottle” for the activity related to a person who walks with a metallic bottle under the jacket, “Limping” for people walking with a limp and “SlowWalk_SwingingHands” for people who walk at slow speed with swinging hands.

**Fig. 4:**
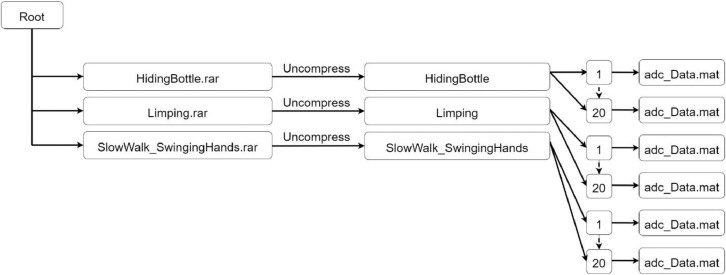
Repository part 2 structure.

As done for repository Part 1, these data are stored inside compressed files. The dataset structure is reported in Fig. 4.

In Table 2 we summarize the measurements contained in dataset_Part2.

**Table 2:** Details of the experiments contained in dataset_Part2.

Activity	N° of subjects	N° of repetitions	Total N° of tests
Walk hiding Bottle	1	20	20
Walk with a limp	10	2	20
Slow walk with swinging hands	10	2	20

### 1.2 Data file description

The dataset contains a collection of files with the “.mat” extension. This type of data is used by Matlab to save variables values inside a workspace. Data can be loaded and processed using Matlab or other languages, such as Python. Since data represent complex signals, they are stored as a complex double data type.

The “adc_Data.mat” files contain a Matlab Table, which is a Matlab variable type [2]. This Table has a dimension of 4 columns by 26,214,400 rows. Each row of the table consists of samples acquired from the 4 Rx antennas at the same sampling time. Each column contains then data coming from a separate receiver so that the column describes the time series of the signal at a particular receiver. The signals related to Rx antenna “n” are called “adcDataRXn” in the Table, where “n” represents the receiver number.

**Fig. 5:**
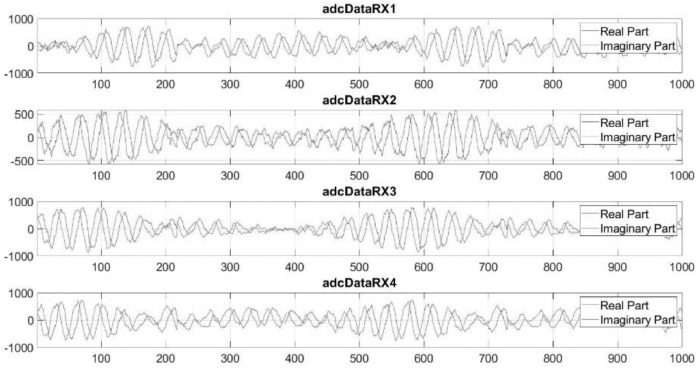
Example of 1000 samples contained within the Matlab table.

Fig. 5 shows an example of 1000 samples collected by 4 different receiving antennas.

The total number of samples within the columns is 26,214,400; for the used configuration this corresponds to an acquisition time of 16 s.

## 2 Experimental design, materials, and methods

The devices exploited to obtain the raw data contained in the repository are the AWR 1642 [3] Radar board and the DCA 1000 EVM [4]. The first board is the radar device, which is a Frequency Modulated Continuous Wave (FMCW) as described in [5]. It is a fully integrated device able to provide the raw beat signal as output. The radar implements MIMO functionalities as described in [6], being equipped with 2 transmitters and four receivers. The dataset here presented is built using only one transmitter and four receivers.

The second board used (DCA 1000 EVM), connected directly to the ADCs of the radar board, extracts the samples of the four beat-signals generated in binary format.

**Fig. 6:**
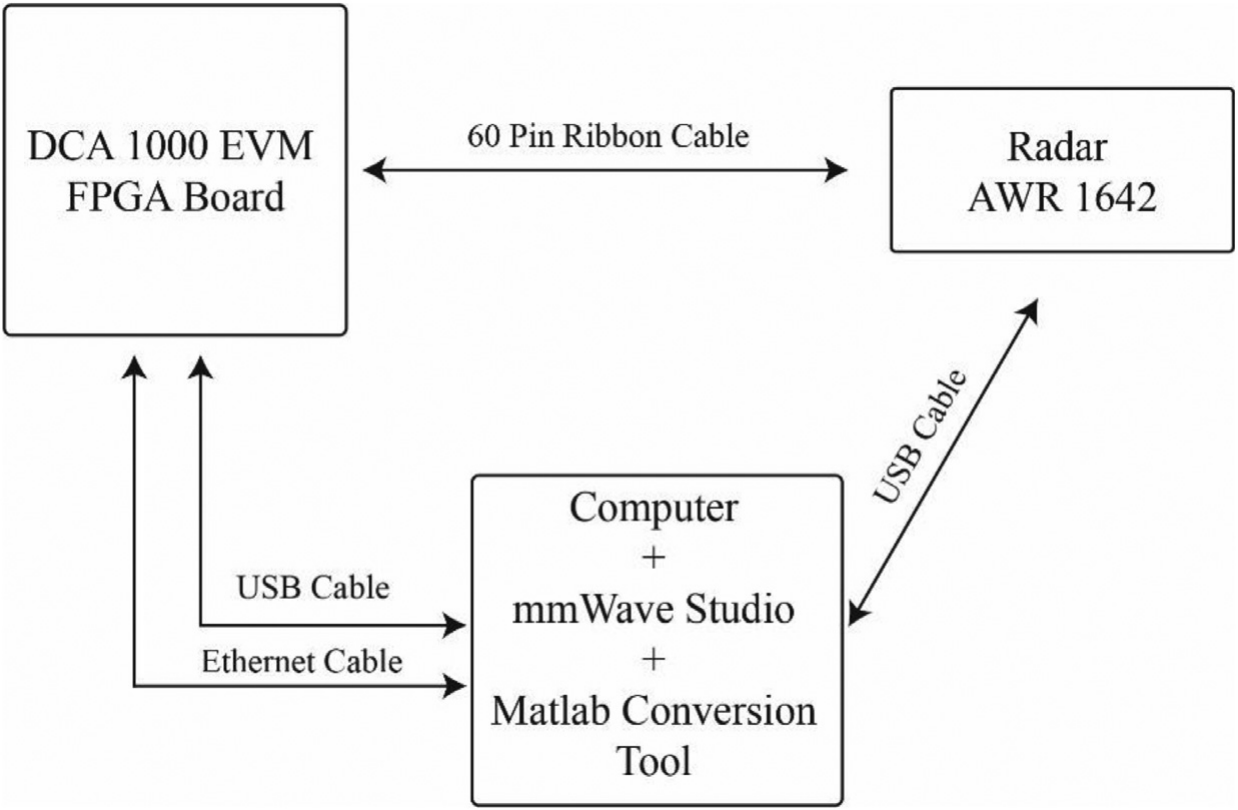
Radar system connection scheme.

A scheme of the connection is depicted in Fig. 6.

Samples collected with the DCA 1000 EVM board are sent via UDP protocol to the computer where the data are stored. By default, these data are stored in a binary file, but in the provided dataset they have been converted to Matlab files with a Matlab Conversion Tool. This conversion does not affect the information content of the files.

Configuration and control of the measurement system are carried out by the mmWave Studio software from Texas Instruments, which provides a graphical interface for all the configuration parameters.

### 2.1 Experiments and radar configuration

The dataset refers to a series of tests made in a hallway of the Information Engineering Department at Marche Polytechnic University. The hallway is 12 m long and is free of furniture. During each experiment, one subject walks inside the test area going away from the radar system and then comes back.

All subjects walk in a natural way without any constriction in order to generate a dataset that is as realistic as possible.

The activity performed are:

• Fast walk;
• Slow walk;
• Slow walk with hands in pockets;
• Walk hiding bottle;
• Walk with a limp;
• Slow walk with swinging hands.

Background acquisition is performed with the same experimental setup, without any human subject in the test field. There is only one file because the same measurement area was used for all the tests.

The configuration of the radar sensor depends on the test performance requirements and on the dimension of the measurement area. This area has a length of about 10 m and we suppose that the speed of the target is lower than 6 m/s.

**Fig. 7:**
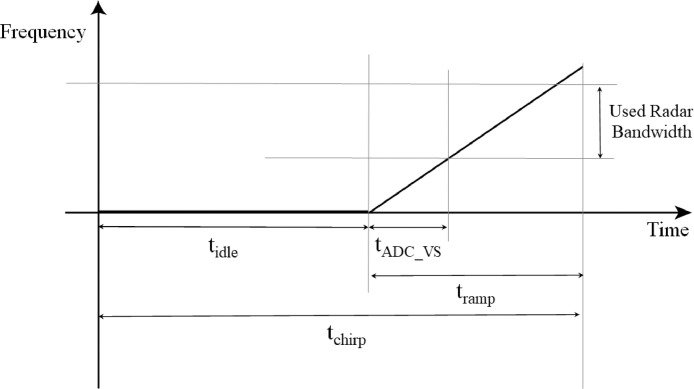
Chirps timing.

The same configuration is chosen for all the performed activities; the detail of parameters is reported in Table 3 and Fig. 7, respectively.

**Table 3:** Radar configuration parameters.

Parameter	Value
Frequency Slope	60.012 MHz/µs
Idle time [*t_idle_*]	100 µs
ADC Valid Start Time [$t_{ADC\_VS}]$	6 µs
Sampling frequency [*f_s_*]	10 Msps
Ramp time [*t_ramp_*]	60 µs
N° of used samples [*n_samples_*]	512
N° of frames [*n_frame_*]	400
N° of chirps per frame [*n_chirps_*]	128
Periodicity	40 ms
Used Radar bandwidth	3.6 GHz

The duration of a single experiment can be computed from this choice of parameters. Each Matlab table contains 4 columns of 26,214,400 samples. Each periodicity interval, which represents the repetition of 128 chirps, contains 65,536 samples. By multiplying this number by the total number of frames, the number of samples of each receiver line is derived.

The Chirp Time *t_chirp_* value can be computed as

$$t_{chirp}=t_{idle}\;+\;t_{ramp},$$

where *t_idle_* is used to wait the restart of the ramp generator. The first part of the ramp is not linear, so a waiting time $t_{ADC\_VS}$, during which the signal is not sampled, is necessary to avoid possible distortions. *t_ramp_* is the configurable duration of the chirp ramp. Accordingly to these definitions, *n_samples_* represents the number of samples within the part of *t_ramp_* effectively used, i.e. $t_{ramp}-t_{ADC\_VS}$.

It follows that the frame time (*t_frame_*) is equal to the Chirp Time multiplied by the number of chirps per frame, or

$$t_{frame\;}=t_{chirp}\;\cdot \;n_{chirps}.$$

In our experiments the frame time is set equal to 20.48 ms. The value of Periodicity is required to be larger than the frame time. In this work, a value of 40 ms for Periodicity is chosen, and each experiment contains 400 frames. It follows that the test total duration is 16 s. This choice of the parameters allows detection of a Doppler shift in the range −8000 Hz to 8000 Hz. The range detection value *R_max_* can be derived as follows [7]. First the beat frequency *f_IF_* is calculated as

$$f_{IF}=\;\frac{2\;R_{max}\cdot \text{Used}\;\text{Radar}\;\text{bandwidth}}{c\cdot \;t_{ramp}},$$

where $c=3\cdot 10^{8}m/s$ represents the speed of light. The maximum detectable value of *f_IF_* is equal to half the value of the sampling frequency *f_s_* of the sensor or, in formulas, $f_{IF}=f_{s}/2$ . The maximum detectable range can be therefore computed as

$$R_{max}=\;\frac{t_{ramp}\cdot c}{4\;\cdot \;\text{Used}\;\text{Radar}\;\text{bandwidth}}\;f_{s}\;=12.4\;m.$$

### 2.2 Data signal pre-processing

The collected data do not come directly from the radar system. As previously stated, samples are sent via UDP from the acquisition board to host computer and stored in a raw binary file which needs a pre-processing before it can be used.

To generate this dataset all binary data are converted through a Matlab Script, that loads the binary file and stores the samples of the beat signals inside a Matlab Table. The Matlab Workspace is then saved in order to provide the adc_data.mat file.

This conversion saves only the samples in a different data arrangement but the raw information is preserved.

## Declaration of Competing Interest

The authors declare that they have no known competing financial interests or personal relationships which have, or could be perceived to have, influenced the work reported in this article.

## Appendix Supplementary materials

Image, application 1
